# Impact of rapid identification by MALDI-TOF MS from positive blood cultures in *Enterococcus* spp. bloodstream infections

**DOI:** 10.1007/s10096-025-05084-x

**Published:** 2025-03-08

**Authors:** Diogo Lopes, Bruno Grandbastien, Christina Orasch, Gilbert Greub, Antony Croxatto, Guy Prod’Hom, Benoit Guery

**Affiliations:** 1 Intensive Care Unit, Curry Cabral Hospital - Central Lisbon University Hospital Centre, Lisboa, Portugal; 2 Infection Control Unit, Service of Infectious Diseases, Lausanne University Hospital and University of Lausanne, Lausanne, Switzerland; 3 Medisyn, Luzern, Switzerland; 4 Institut of Microbiology, Lausanne University Hospital and University of Lausanne, Lausanne, Switzerland; 5 Microbiologie, ADMED, La Chaux-de-Fonds, Switzerland; 6 Service of Infectious Diseases, Lausanne University Hospital and University of Lausanne, Lausanne, Switzerland

**Keywords:** *Enterococcus*, Bloodstream infection, Bacteraemia, Gram-positive bacteria, MALDI-TOF

## Abstract

**Purpose:**

Regarding bloodstream infections (BSI) *Enterococcus* spp. rank among the top five most common organisms. Due to enterococci intrinsic resistance, empiric antibiotic therapy is often inappropriate and early identification becomes crucial. Our objective was to assess the clinical impact of MALDI-TOF identification directly from positive blood cultures (BC) in *Enterococcus* spp. BSI (E-BSI).

**Methods:**

A retrospective cohort study included all adult patients with E-BSI from 2010 to 2017 in a tertiary hospital. ID consultation within 48 h and MALDI-TOF identification directly from BC within 24 h were inclusion criteria. The primary outcome was antimicrobial treatment change following MALDI-TOF and secondary outcomes included 30-day and 1-year mortality, length of stay (LOS) and antimicrobial de-escalation.

**Results:**

Among 267 BSI episodes, *E. faecalis* was isolated in 130 episodes (48.7%), *E. faecium* in 122 (45.7%), and 104 (39%) were polymicrobial. Empiric antibiotic therapy was inappropriate in 60.3% of patients. The LOS was 36 (IQR 20–64) days, 30-day and 1-year mortality were 16.1% and 43.4%, respectively. Enterococci identification with MALDI-TOF at the species level was possible in 66.3% cases and in 73% of monomicrobial cases. Antibiotics were changed in 85.3% of the former vs. 63.3% in remaining patients (*p* < 10^− 4^), and de-escalation occurred in 35% of subjects (vs. 12.2%,*p* = 10^− 4^). Changing antibiotics after correct identification was associated with a shorter LOS. In multivariate analysis, appropriate antibiotic therapy before MALDI-TOF was protective against 30-day mortality (aOR 0.40(0.08–1.96)), and appropriate antibiotic therapy afterwards against 1-year mortality (aOR 0.21(0.05–0.84)).

**Conclusion:**

In E-BSI, direct MALDI-TOF identification from positive BC has a significant clinical impact due to a more frequent antibiotic spectrum correction and de-escalation. This may improve patient outcomes, reducing LOS and potentially mortality.

**Clinical trial number:**

Not applicable.

**Supplementary Information:**

The online version contains supplementary material available at 10.1007/s10096-025-05084-x.

## Introduction

*Enterococcus* spp. rank second among all adult healthcare-associated infections in the United States [1]. Regarding bloodstream infections (BSI) enterococci are one of the top five most common organisms [2], with a proportion up to 28% in critically ill patients with hospital-acquired BSI [3]. Furthermore, the incidence is increasing [4] and mortality remains high, reaching up to 51% [5–10].

Negative outcomes due to delays in appropriate antimicrobial therapy are well-established [11–13], prompting guidelines to emphasize the rapid initiation of antimicrobials in patients with a high likelihood of sepsis [14]. However, empiric antimicrobial therapy proves inappropriate in 18 to 48% of patients [15–18]. Enterococci are intrinsically resistant to many antibiotics, particularly most cephalosporins and aminoglycosides. *E. faecium* is generally more resistant than *E. faecalis*, with most strains intrinsically resistant to other beta-lactams and sometimes to vancomycin. In a large study on community-onset sepsis, 43% of patients were on cephalosporins as empiric antimicrobial therapy [16]. Another retrospective study on BSI showed *Enterococcus* spp. to be the second most powerful predictor of inappropriate antibiotic therapy, with inadequacy rates in up to 75% [18]. This is associated with poor outcomes, including a longer length of stay (LOS) [19] and increased mortality [16, 18, 19], also in enterococcal BSI (E-BSI) [6, 20].

Currently, matrix-assisted laser desorption/ionization time-of-flight mass spectrometry (MALDI-TOF) is the most commonly used approach for rapid pathogen identification directly from positive blood cultures (BC) [21], allowing a reduction in the time to microorganism identification, time to appropriate antibiotic therapy, LOS, mortality, and costs [21]. High accuracy in enterococci identification by MALDI-TOF has been frequently reported [22]. However, there is limited data evaluating its clinical impact specifically on patients with E-BSI. Only one retrospective study with an assessment of 91 isolates in the MALDI-TOF group observed a shorter time to definitive antibiotic treatment with similar 28-day mortality and LOS [9].

The aim of our study was thus to assess the clinical impact of MALDI-TOF identification directly from positive BC on the clinical management of patients with E-BSI.

## Materials and methods

### Design

**Fig. 1:**
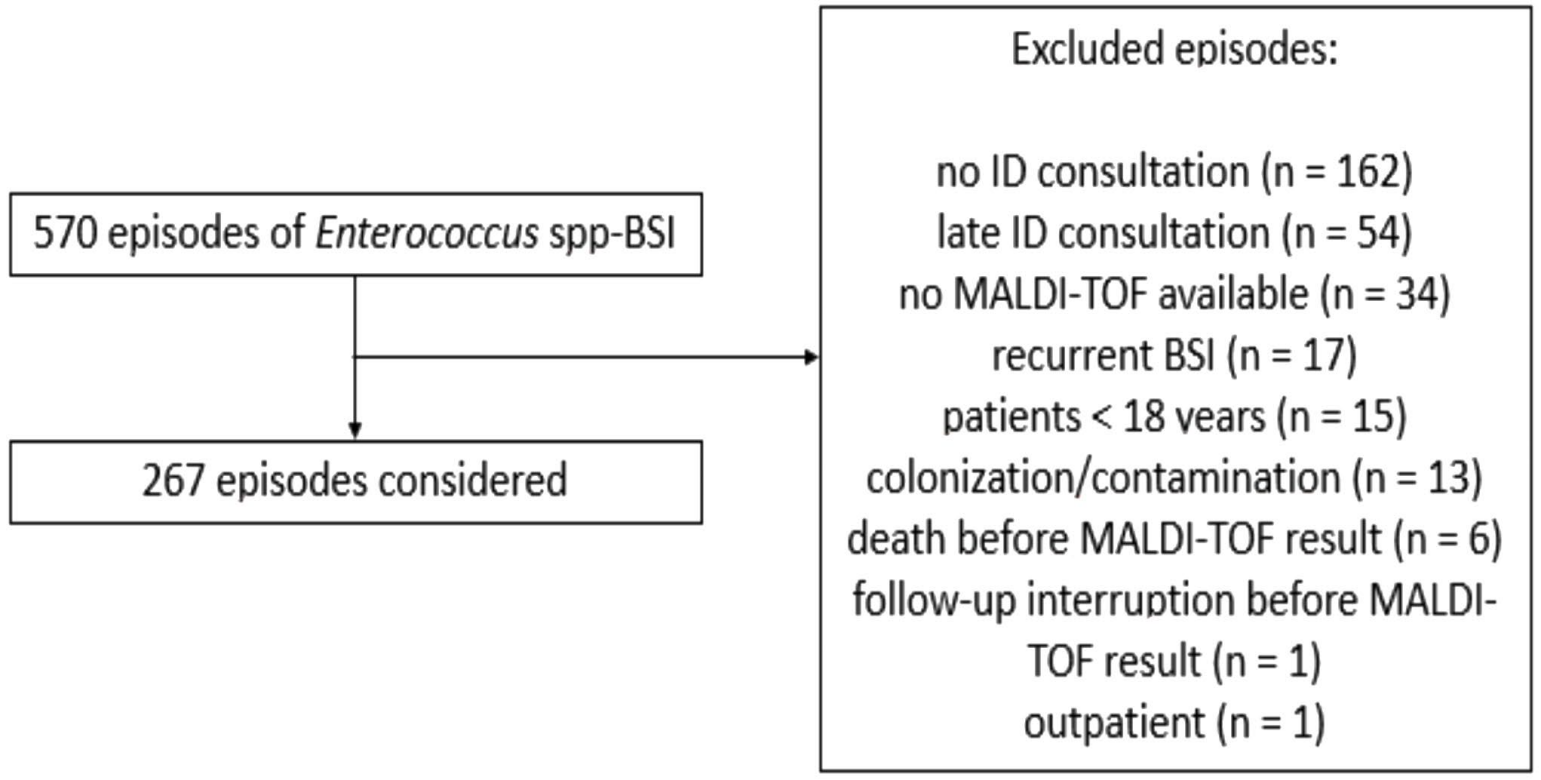
Flowchart of *Enterococcus* spp. bloodstream episodes studied

We conducted a retrospective study encompassing all patients with E-BSI from 2010 to 2017 at the University Hospital of Lausanne. Inpatients experiencing their initial episode of E-BSI were included. Exclusions comprised patients under 18 years old, MALDI-TOF results later than 24 h since BC collection in patients, absence of MALDI-TOF results, absence of an infectious disease (ID) consultation within 48 h, colonization or contamination as per ID consultant’s judgment, follow-up interruption, or death before MALDI-TOF identification (Fig. 1).

The primary outcome was a change in antimicrobial treatment following the MALDI-TOF result. Secondary outcomes included 30-day and 1-year mortality (time period accounted from E-BSI date), LOS, and antimicrobial de-escalation.

Baseline characteristics, clinical and microbiological data of patients were extracted from electronic medical records. Data collection concluded in July 2019, ensuring a complete 1.5-year follow-up for all patients.

### Definitions

E-BSI was defined by the isolation of *Enterococcus* spp. from one or more sets of aseptically obtained BC bottles. A single episode was considered per patient. If another clinically relevant agent was present in the BC, including enterococci, BSI was classified as polymicrobial. Immunosuppression included neutropenia (neutrophil count < 500/mm^3^ for ≥ 7 days), solid-organ transplantation, hematopoietic stem cell transplantation, active hematologic malignancy, solid malignancy under chemotherapy or immunotherapy during the last 6 months, primary immunodeficiency, and ≥ 20 mg of prednisone per day or equivalent longer than 30 days. Infections were considered nosocomial when diagnosed more than 48 h after hospital admission and healthcare-associated within 48 h after admission if another hospitalization occurred within 90 days before the onset of BSI, or if the patient came from a long-term facility. Septic shock was defined according to the Sepsis-3 definition [23].

Antimicrobial treatment was classified as *appropriate* if the *Enterococcus* strain isolated from BC was susceptible to at least one antibiotic administered when MALDI-TOF identification was reported. *De-escalation* was achieved by narrowing the antimicrobial spectrum or by switching from combination antibiotics to monotherapy [24]. Classification of all antibiotic decisions is presented in Tables S1 and S2, following previous orientations regarding spectrum ranking of beta-lactams [25, 26]. Treatment was considered *targeted* after MALDI-TOF identification result if amoxicillin/penicillin were chosen for enterococcal strains sensitive to these antibiotics or vancomycin/daptomycin for resistant ones. The addition of ceftriaxone for endocarditis or synergetic gentamicin on severe disease was not considered a modification of antibiotic therapy or spectrum, nor was non-targeted therapy. Classification regarding appropriateness or spectrum of antibiotic therapy was based on decisions made within 24 h after the MALDI-TOF identification.

### Routine procedures

Positive BC were detected using the BACTEC FX 9240 automated BC system (Becton Dickinson, Sparks, MD). Gram staining was promptly performed on all positive BC between 8 AM and 7 PM, and early the following morning when they became positive overnight. Direct MALDI-TOF has been routinely conducted in our center since September 2009 on all positive BC immediately after Gram staining. An ammonium chloride erythrocyte-lysing procedure is employed to prepare a bacterial pellet from positive BC before MALDI-TOF, as previously described [27, 28]. The protein extraction was done directly on the MALDI-TOF microplate, by mixing a bacterial colony obtained by culture with formic acid [29]. Mass spectra were acquired using a Microflex LT MALDI-TOF instrument (Bruker Daltonics, Germany). Spectral analysis and comparison with the database were executed using MALDI BioTyper 2.0 software. Following the criteria proposed by the manufacturer, identification was considered reliable at the species and at the genus level when the score was ≥ 2 and and between 1.7 and 2, respectively. Antimicrobial susceptibility testing was directly performed from the blood culture pellet (enrichment and purification) with the Vitek^®^ 2 system and/or with disk diffusion, or from colonies the day after. European Committee on Antimicrobial Susceptibility Testing (EUCAST) and Clinical and Laboratory Standards Institute (CLSI) were the references for clinical breakpoints.

Gram stain and MALDI-TOF-based identification results were reported by clinical microbiologists to the clinician in charge approximately 1 to 2 h after the positivity of the BC and directly to ID clinicians during a daily meeting. Clinicians overseeing the patients requested ID consultations as necessary, assuming the final decision on patient management.

### Ethics

This study received approval from hospital Ethics Committee (n° 2019 − 00742). Patient consent was waived, as this is a retrospective cohort study.

### Statistics

Categorical variables were compared using the χ^2^ or Fisher exact tests and continuous variables using the Mann-Whitney test. Survival differences were evaluated using a Kaplan-Meier curve with log-rank test. A multivariate analysis of 30-day and 1-year mortality was performed by a logistic regression model (backward stepwise method) to examine predictors by adjusted odds ratios (aOR) with 95% confidence intervals (95% CI). Mortality was adjusted for age, gender, LOS, day of BSI, nosocomial or healthcare-associated, comorbidities, immunosuppression, presence of shock, Intensive Care Unit (ICU) admission, enterococci strain, polymicrobial, source of BSI and antibiotic decision and spectrum. A *p*-value < 0.05 was considered statistically significant. Analyses were conducted on EZR^®^, version 1.61, and on IBM^®^ SPSS^®^ Statistics software, version 29.

## Results

During the study period, a total of 570 patients with positive BC for *Enterococcus* spp. were identified, and 267 were included in the final analysis (Fig. 1). Most patients were male (69.7%) with a median age of 71 (interquartile range 60–78) years and Charlson comorbidity index of 6 (IQR 4–7). Past or active solid malignancy was the most prevalent comorbidity (40.4%), primarily involving the gastrointestinal tract (pancreas and biliary tract). Comparison of comorbidities between patients with *E. faecalis* and *E. faecium* BSI is presented in Table 1.

**Table 1:** Characteristics of patients with *E. faecalis* or *E. faecium* bloodstream infection (15 patients with non-faecalis or non-faecium *Enterococcus* spp. were not stratified due to low “n”)

Characteristics of patients	*Enterococcus* spp. *N* = 267 (IQR or %)	*E. faecalis* *n* = 130 (IQR or %)	*E. faecium* *n* = 122 (IQR or %)	*p*-value*
Age (years)	71 (60–78)	71 (62–80)	70 (58–78)	0.399
Sex (male)	186 (69.7)	99 (76.2)	75 (61.5)	**0.017**
Charlson comorbidity index	6 (4–7)	6 (4–7)	6 (4–7)	0.913
Comorbidities				
Cardiomyopathy	85 (31.9)	53 (40.8)	26 (21.3)	**0.001**
Chronic pulmonary disease	41 (15.4)	21 (16.2)	15 (12.3)	0.487
Cirrhosis	24 (9.0)	7 (5.4)	14 (11.5)	0.128
Chronic kidney disease (stage III-V)**	48 (18.0)	27 (20.8)	19 (15.6)	0.366
Dialysis	15 (5.6)	10 (7.7)	5 (4.1)	0.291
Diabetes	60 (22.5)	35 (26.9)	22 (18)	0.125
Immunosuppression	79 (29.6)	35 (26.9)	43 (35.2)	0.196
Transplantation	21 (7.9)	11 (8.5)	10 (8.2)	1.000
Haematological malignancy	28 (10.5)	14 (10.8)	13 (10.7)	1.000
Solid malignancy***	108 (40.4)	47 (36.2)	57 (46.7)	0.097
Gastrointestinal tract	64 (24.0)	22 (16.9)	40 (32.8)	**0.006**
Urinary tract	24 (9.0)	13 (10.0)	9 (7.4)	0.621
Other	30 (11.2)	17 (13.1)	12 (9.8)	0.543
Septic shock	33 (12.4)	13 (10.0)	16 (13.1)	0.564
Intensive Care Unit admission	43 (16.1)	19 (14.6)	19 (15.6)	0.971
Clinical source of BSI				
Gastrointestinal tract	103 (38.6)	24 (18.5)	67 (54.9)	**< 0.001**
Urinary tract	40 (15.0)	31 (23.8)	9 (7.4)	**< 0.001**
Catheter-related	34 (12.7)	21 (16.2)	13 (10.7)	0.275
Other (surgical site, soft tissue, other endovascular, osteoarticular)	29 (10.9)	16 (12.3)	12 (9.8)	0.672
Endocarditis	18 (6.7)	15 (11.5)	3 (2.5)	**0.006**
Unknown (primary)	43 (16.1)	23 (17.7)	18 (14.8)	0.645
Nosocomial BSI	180 (67.4)	78 (60.0)	95 (77.9)	**0.004**
Healthcare-associated BSI	51 (19.1)	32 (24.6)	17 (13.9)	**0.048**
Days until BSI since admission	11 (1–29)	9 (0–24)	17 (5–31)	**0.006**
Polymicrobial BSI	104 (39)	53 (40.8)	42 (34.4)	0.364
Inappropriate antibiotic therapy before MALDI-TOF	161 (60.3)	60 (46.2)	91 (74.6)	**< 10** ^**− 5**^
Targeted antibiotic therapy before MALDI-TOF	4 (1.5)	2 (1.5)	2 (1.6)	1.000
Outcome				
Hospital length of stay (days)	36 (20–64)	35 (16–57)	41 (26–69)	**0.034**
D30 mortality	43 (16.1)	13 (10.0)	27 (22.1)	**0.014**
1-year mortality	116 (43.4)	47 (36.2)	63 (51.6)	**0.019**

Abbreviations: BSI, bloodstream infection; IQR, interquartile range;

* *p*-value regards comparison between *E. faecalis* and *E. faecium*;

**Chronic kidney injury according to KDIGO classification;

***Cured or active malignancy were considered

Among all episodes, 130 (48.7%) were attributed to *E. faecalis* and 122 (45.7%) to *E. faecium* (15% of strains were amoxicillin-sensitive). Fifteen non-faecalis non-faecium enterococci were identified (5.6%) (Table S3). One *E. faecium* and three non-faecalis non-faecium enterococci isolates were resistant to vancomycin, while the latter were amoxicillin-sensitive.

More than two-thirds of infections were nosocomial (67.4%), and healthcare-associated BSI accounted for 19.1%, both more commonly observed in *E. faecium* BSI (Table 1). The median day of BSI was day 11 (IQR 1–29) after admission, with a later onset for *E. faecium* compared to *E. faecalis*.

Prior to MALDI-TOF analysis, the antibiotic spectrum was inappropriate in 161 patients (60.3%), particularly in *E. faecium* compared to *E. faecalis* (74.6% vs. 46.2%, *p* < 10^− 5^). Contrarly, inadequacy was less prevalent in patients in shock or admitted to ICU comparing to remaining ones (39.4% vs. 63.2%, *p* = 0.015, and 39.5% vs. 64.3%, *p* = 0.004, respectively).

39% (n = 104) of BSI were polymicrobial. BC with non-faecalis non-faecium enterococci were more frequently polymicrobial (60.0%) than those with *E. faecalis* (40.8%) (*p* = 0.25) and *E. faecium* (34.4%) (*p* = 0.099). Polymicrobial BSI were more prevalent in cases originating from ‘other source’ (58.6%), the gastrointestinal tract (42.7%), and an unknown source (41.9%). At least one Gram-negative bacterium was present in 67.3%, a Gram-positive in 45.2%, another *Enterococcus* species in 15.4% and *Candida* spp. in 7.7% of polymicrobial cases.

Overall, a MALDI-TOF identification with a score ≥ 2 was present in 73.0% (*n* = 195), and a score ≥ 1.7 in 87.3% (*n* = 233) of BC. Regardless of the score, *Enterococcus* spp. were identified with MALDI-TOF in 83.5% of all episodes, rising to 92.6% if we exclude polymicrobial BSI (Table 2). At the species level the identification of *Enterococcus* spp. was possible in 66.3% of episodes and at the genus level in 78.3%, more frequently in monomicrobial BSI compared to polymicrobial. The lowest performance was found in non-faecalis non-faecium enterococci with identification in only 53.3% of episodes. A trend to a better performance of MALDI-TOF on enterococci identification at the species level has been observed comparing isolates between 2010 and 2013 and 2014–2017 (58.0% vs. 70.4%, *p* = 0.06).

**Table 2:** **–** Identification of *Enterococcus* spp. on matrix-assisted laser desorption/ionization time-of-flight (MALDI-TOF)

MALDI-TOF identification score		*N* = 267 (%)	*E. faecalis* *n* = 130 (48.6)	*E. faecium* *n* = 122 (45.7)	Non-faecalis non-faecium enterococci *n* = 15 (5.6)	*p*-value*	Monomicrobial *n* = 163 (61.0)	Polymicrobial *n* = 104 (39.0)	*p*-value	Polymicrobial (≥ 1 Gram-) *n* = 70 (26.2)	Polymicrobial (≥ 1 Gram+) *n* = 47 (17.6)	*p*-value
***Enterococcus*** **spp.**	**Any**	223 (83.5)	113 (86.9)	102 (83.6)	8 (53.3)	0.004**	151 (92.6)	72 (69.2)	**< 0.001**	41 (58.6)	39 (83.0)	**< 0.010**
	**≥ 2**	177 (66.3)	90 (69.2)	80 (65.6)	7 (46.7)	0.211	119 (73.0)	58 (55.8)	**0.006**	34 (48.6)	29 (61.7)	0.227
	**1.7 ≥ x > 2**	32 (12.0)	14 (10.8)	17 (13.9)	1 (6.7)	0.667	23 (14.1)	9 (8.6)	0.246	4 (5.7)	7 (14.9)	0.115
	**≥ 1.7**	209 (78.3)	104 (80.0)	97 (79.5)	8 (53.3)	0.054***	142 (87.1)	67 (64.4)	**< 0.001**	38 (54.3)	36 (76.6)	**0.024**
**Non-** ***Enterococcus***	**Any**	44 (16.5)	17 (13.1)	20 (16.4)	7 (46.7)	**0.004**	12 (7.4)	32 (30.8)	**< 0.001**	29 (41.4)	8 (17.0)	**< 0.010**
	**≥ 2**	18 (6.7)	7 (5.4)	8 (6.6)	3 (20.0)	0.116	0 (0.0)	18 (17.3)	**< 0.001**	16 (22.9)	4 (8.5)	**0.048**
	**1.7 ≥ x > 2**	6 (2.2)	2 (1.5)	3 (2.5)	1 (6.7)	0.363	0 (0.0)	6 (5.8)	**0.003**	6 (8.6)	1 (2.1)	0.240
	**≥ 1.7**	24 (9.0)	9 (6.9)	11 (9.0)	4 (26.7)	0.055	0 (0.0)	24 (23.1)	**< 0.001**	22 (31.4)	5 (10.6)	**0.017**

* *p-value regards comparison between E. faecalis*,* E. faecium* and non-faecalis non-faecium enterococci;

**p < 0.05 between *E. faecalis* or *E. faecium* and non-faecalis enterococci;

***p < 0.05 between *E. faecalis* and non-faecalis non-faecium enterococci, *p* = 0.053 between *E. faecium* and non-faecalis non-faecium enterococci

Concerning those 44 isolates other than *Enterococcus* spp., 40.9% had a score ≥ 2, and 54.5% had a score ≥ 1.7, all of which were detected in polymicrobial BSI (Table 2).

After enterocci identification with a score ≥ 2 in MALDI-TOF, antibiotic therapy changed in 85.3% of episodes against 63.3% when the score was < 2, similarly to the difference found in spectrum appropriatess (99.4% vs. 83.3%) (both with *p* < 10^− 4^) (Table 3). Adjustment was also more frequent in cases of inappropriate empiric therapy compared to patients with ongoing enterococci coverage (99.0% vs. 67.5%, *p* < 0.001) and non significant in monomicrobial cases vs. polymicrobial. (Table 3).

**Table 3:** Early antibiotic decision according to Matrix-assisted laser desorption/ionization time-of-flight (MALDI-TOF) identification score, according to enterococci strains, the presence of other organisms and appropriateness of antibiotic therapy before MALDI-TOF

Early antibiotic decision after MALDI-TOF identification			Change		De-escalation		Appropriate		Targeted	
		**N**	n (%)	***p*** **-value**	n (%)	***p*** **-value**	n (%)	***p*** **-value**	n (%)	***p*** **-value**
**All isolates**		267	208 (77.9)		73 (27.3)		251 (94.0)		77 (28.8)	
**MT ENT ID score ≥ 2**		177	151 (85.3)	**< 0.0001**	62 (35.0)	**0.0001**	176 (99.4)	**< 0.0001**	59 (33.3)	**0.033**
**MT ENT ID score < 2**,** no ID or other ID**		90	57 (63.3)		11 (12.2)		75 (83.3)		18 (20.0)	
**MT ENT ID score 1.7 ≥ x > 2**		32	27 (84.4)	1.000*	8 (25.0)	0.367*	30 (93.8)	0.062*	12 (37.5)	0.799*
**MT ENT ID score ≥ 1.7**		209	178 (85.2)	**< 0.0001**	70 (33.5)	**< 0.0001**	206 (98.6)	**< 0.0001**	71 (34.0)	**< 0.001**
**MT ENT ID score < 1.7**,** no ID or other ID**		58	30 (51.7)		3 (5.2)		45 (77.6)		6 (10.3)	
**MT ID** ***Enterococcus*** **score ≥ 2 (n = 177)**	**E.** ***faecalis***	90	75 (83.3)	0.745	44 (48.9)	**< 0.001**	89 (98.9)	0.615	41 (45.6)	**0.002**
	**E.** ***faecium***	80	70 (87.5)		16 (20.0)		80 (100.0)		17 (21.2)	
	**Non-faecalis non-faecium**	7	6 (85.7)		2 (28.6)		7 (100.0)		1 (14.3)	
	**Monomicrobial**	119	106 (89.1)	0.072	50 (42.0)	**0.009**	119 (100.0)	0.713	48 (40.3)	**0.008**
	**Polymicrobial**	58	45 (77.6)		12 (20.7)		57 (98.3)		11 (19.0)	
	**Appropriate antibiotic before MT**	77	52 (67.5)	**< 0.001**	38 (49.4)	**0.001**	77 (100.0)	1.000	22 (28.6)	0.308
	**Inappropriate antibiotic before MT**	100	99 (99.0)		24 (24.0)		99 (99.0)		37 (37.0)	

* - *p*-value was obtained comparing patients with MALDI-TOF identification score between 1.7 and 2 (genus level) to those with score ≥ 2 (species level)

Abbreviations: ID, identification; ENT, *Enterococcus* spp.; MT, matrix-assisted laser desorption/ionization time-of-flight;

De-escalation happened in 35% of patients with identification at the species level vs. 12.2% in remaining patients (*p* = 10^− 4^). Appropriate empiric therapy, *E. faecalis* episodes, from urological source, monomicrobial and non-nosocomial were also associated to de-escalation (Table 3 and Table S4). Likewise, targeted therapy after MALDI-TOF was also more prevalent in *E. faecalis* and monomicrobial BSI, and additionally in earlier episodes (9 (IQR 0–23) vs. 15 (IQR 2–30) days, *p* = 0.09) and endocarditis. In contrast, in gastrointestinal source or shock this was less frequent.

**Fig. 2:**
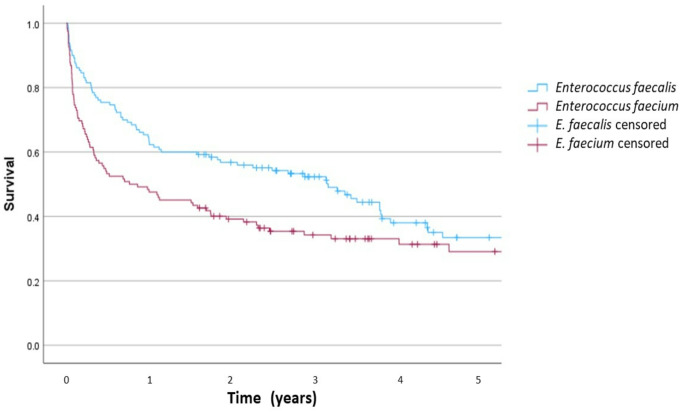
− 5-year survival among patients with *E. faecalis* or *E. faecium* bloodstream infection determined by Kaplan–Meier curve (*p* = 0.028)

The median in-hospital stay was 36 (IQR 20–64) days, being significantly longer LOS in *E. faecium* BSI compared to *E. faecalis* (Table 1). Mortality at 30 days and 1 year was 16.1% and 43.4%, respectively (Table 1). In comparison to *E. faecalis* episodes, patients with *E. faecium* BSI died more frequently (10.0% vs. 22.1%, *p* = 0.014, at 30 days and 36.2 vs. 51.6, *p* = 0.019 at 1 year) (Table 1; Fig. 2) and non-faecalis non-faecium enterococci were in between. The 30-day and 1-year mortality according to clinical characteristics is in Table S5.

Changing antibiotics and de-escalation after MALDI-TOF were associated with a shorter LOS compared to those who did not, like patients with targeted therapy (Table 4). Patients with inappropriate antibiotic therapy prior to and after the MALDI-TOF identification had a higher, albeit non-significant, 30-day mortality (19.3% vs. 11.3%, *p* = 0.09, and 25.0% vs. 15.5%, *p* = 0.48) and 1-year mortality, as did those where de-escalation was not performed or without targeted antibiotic therapy (Table 4). Those whose antibiotics were altered died more frequently compared to those without a change (not statistically significant).

**Table 4:** Outcomes according to antibiotic decision after Matrix-assisted laser desorption/ionization time-of-flight (MALDI-TOF) result in isolates with identification of an *Enterococcus* spp. (*n* = 223), and according to antibiotic therapy before and after MALDI-TOF result in all patients (*n* = 267)

MT ID *Enterococcus* spp. *N* = 223	Antibiotic change, *n* (IQR or %)						De-escalation, *n* (IQR or %)					
	score ≥ 1.7			score ≥ 2			score ≥ 1.7			score ≥ 2		
	Yes 178 (79.8)	No 31 (13.9)	*p*-value	Yes 151 (67.7)	No 26 (11.7)	*p*-value	Yes 70 (31.4)	No 139 (62.3)	*p*-value	Yes 62 (27.8)	No 115 (51.6)	*p*-value
**Hospital LOS (days)**	36 (20–57)	71 (32–129)	**0.002**	36 (18–59)	62 (34–130)	**0.004**	33 (17–49)	39 (23–75)	**0.060**	33 (17–49)	39 (24–80)	**0.036**
**30-day mortality**	31 (17.4)	2 (6.5)	0.181	27 (17.9)	2 (7.7)	0.259	8 (11.4)	25 (18.0)	0.305	7 (11.3)	22 (19.1)	0.258
**1-year mortality**	84 (47.2)	13 (41.9)	0.729	71 (47.0)	10 (38.5)	0.551	29 (41.4)	68 (48.9)	0.380	24 (38.7)	57 (49.6)	0.221

All BSI *N* = 267	Antibiotic prior to MALDI-TOF result, n (IQR or %)						Antibiotic after MALDI-TOF result, n (IQR or %)					
	Appropriate			Targeted			Appropriate			Targeted		
	Yes 106 (39.7)	No 161 (60.3)	*p*-value	Yes 4 (1.5)	No 263 (98.5)	*p*-value	Yes 251 (94.0)	No 16 (6.0)	*p*-value	Yes 77 (28.8)	No 190 (72.2)	*p*-value
**Hospital LOS (days)**	40 (21–73)	34 (17–62)	0.106	43 (31–103)	36 (20–64)	0.504	36 (20–62)	33 (22–66)	0.882	31 (18–48)	38 (20–70)	**0.049**
**30-day mortality**	12 (11.3)	31 (19.3)	0.090	0 (0.0)	43 (16.3)	1.000	39 (15.5)	4 (25.0)	0.480	9 (11.7)	34 (17.9)	0.286
**1-year mortality**	41 (38.7)	75 (46.6)	0.251	0 (0.0)	116 (44.1)	0.135	105 (41.8)	11 (68.8)	**0.065**	29 (37.7)	87 (45.8)	0.281

Abbreviations: BSI, bloodstream infection; ID, identification; IQR, interquartile range; LOS, length of stay; MT, MALDI-TOF

In the multivariate analysis of 30-day mortality, an increased risk of death was observed with ICU admission, immunosuppression, cirrhosis, endocarditis, catheter-related infections, unknown source, gastrointestinal source, and polymicrobial BSI (Table 5). Healthcare-associated BSI and appropriate antibiotic therapy before MALDI-TOF were associated with a lower risk of death. An increased risk for 1-year mortality was observed with higher Charlson comorbidity index, cirrhosis, transplantation, active hematologic malignancy, non-gastrointestinal non-urological malignancy, shock, and gastrointestinal source of BSI. Appropriate antibiotic therapy after MALDI-TOF was associated with long-term survival.

**Table 5:** Multivariate analysis of mortality at day 30 and 1 year (only variables with *p* < 0.10 on univariate or multivariate analysis are shown)

	30-day mortality				1-year mortality			
	OR (95% CI)	*p*-value	aOR (95% CI)	*p*-value	OR (95% CI)	*p*-value	aOR (95% CI)	*p*-value
Age	1.03 (0.99–1.08)	0.152	1.04 (0.99–1.08)	0.064				
Hospital length of stay	0.97 (0.95–0.99)	**0.002**	0.98 (0.96–0.99)	**0.005**				
BSI day	1.03 (1.01–1.05)	**0.003**	1.03 (1.01–1.04)	**0.004**	1.01 (0.99–1.02)	0.467	1.01 (1.00-1.02)	0.09
Healthcare-associated BSI	0.24 (0.03–2.20)	0.205	0.12 (0.03–0.57)	**0.008**				
Charlson comorbidity index	1.38 (1.04–1.84)	**0.026**	1.21 (0.99–1.48)	0.062	1.34 (1.10–1.63)	**0.004**	1.37 (1.19–1.56)	**< 0.001**
Immunosuppression	2.06 (0.28–15.26)	0.480	2.84 (1.00-8.04)	**0.049**				
Steroids	0.17 (0.01–2.98)	0.226	0.15 (0.16–1.41)	0.098				
Hematologic malignancy (active)					3.17 (0.8–12.30)	0.095	3.98 (1.52–10.41)	**0.005**
Transplantation					2.82 (0.48–16.61)	0.251	3.48 (1.16–10.47)	**0.027**
Intesive care unit	24.00 (4.28–134.6)	**< 0.001**	17.7 (5.09–61.62)	**< 0.001**				
Shock					2.48 (0.74–8.27)	0.141	3.11 (1.25–7.77)	**0.015**
Cardiomyopathy	0.38 (0.10–1.43)	0.153	0.34 (0.11–1.09)	0.069	0.41 (0.17–0.98)	**0.046**	0.54 (0.26–1.12)	0.099
Cirrhosis	11.34 (2.09–61.7)	**0.005**	10.34 (2.43–43.99)	**0.002**	4.17 (1.30-13.33)	0.016	3.09 (1.09–8.74)	**0.033**
Gastro-intestinal malignancy	5.00 (0.31–81.47)	0.258	2.96 (0.98–8.92)	0.054	1.50 (0.23–9.71)	0.668	2.04 (0.97–4.28)	0.060
Urological malignancy	0.12 (0.01–3.06)	0.201	0.09 (0.01–1.11)	0.060				
Non-GI, non-urological malignancy					1.68 (0.27–10.3)	0.574	2.74 (1.06–7.06)	**0.037**
Polymicrobial	9.2 (2.78–30.5)	**< 0.001**	6.36 (2.27–17.82)	**< 0.001**				
Gastro-intestinal source	10.11 (1.16–88.4)	**0.036**	12.87 (1.77–93.21)	**0.011**	3.23 (1.02–10.22)	**0.047**	1.8 (0.98–3.36)	**0.037**
Urological source	9.15 (0.48–176.5)	0.142	9.40 (0.70-125.73)	0.090	3.34 (0.81–13.86)	0.097		
Endocarditis	144.2 (6.83-3042.8)	**0.001**	125.04 (7.34-2131.32)	**< 0.001**				
Unknown source	12.43 (1.04-148.55)	**0.046**	12.01 (1.28-112.49)	**0.029**				
Catheter-related	15.84 (1.38-181.66)	**0.026**	18.53 (2.02-170.04)	**0.010**				
Appropriate antibiotic before MT	0.40 (0.08–1.96)	0.259	0.35 (0.12–0.99)	**0.047**				
Antibiotic change after MT					1.97 (0.65–6.01)	0.232	2.10 (0.92–4.77)	0.078
Appropriate antibiotic after MT					0.24 (0.05–1.21)	0.084	0.21 (0.05–0.84)	**0.027**

Abbreviations: aOR, adjusted odds ratio; BSI, bloodstream infection; OR, odds ratio estimate; CI, confidence interval; GI, gastro-intestinal; MT, MALDI-TOF

## Discussion

To our knowledge, this is the largest cohort study on MALDI-TOF performance and its clinical impact on antibiotic therapy for E-BSI. A MALDI-TOF identification from positive BC of *Enterococcus* spp. at the species level triggered a change in antibiotic therapy in 85% of patients compared to 63% in the remaining patients. This is of paramount importance, particularly when observing that this proportion augmented to 99% in patients who had received previous inappropriate treatment. Overall, considering all isolates, the impact could be even greater since 60% of our entire cohort had inadequate empiric antibiotic therapy. Even with a very low prevalence of multidrug-resistant bacteria, this data confirms the high proportion of inadequacy observed in previous series [18]. Knowing the association between early appropriate therapy and survival in E-BSI [6, 20], which we have also observed, these findings highlight the relevance of rapid MALDI-TOF identification from positive BC in E-BSI.

Another example of MALDI-TOF impact in E-BSI was that after an identification at the species level, antibiotic therapy was more frequently appropriate than in the remaining patients (99% vs. 83%, *p* < 10^− 4^). Knowing MALDI-TOF is faster than traditional methods, this potentially improves time to appropriate therapy, unanimously reported in the literature [21].

Changing antibiotics after correct MALDI-TOF identification was associated with a shorter LOS, similarly to previous works [21], but not yet described in E-BSI [9]. Appropriate antibiotic therapy after MALDI-TOF was independently associated with 1-year survival, as previously suggested [21].

Furthermore, after an identification at the species level, early de-escalation occurred in 35% (49% considering specifically *E. faecalis*) against 12% in remaining patients and antibiotic therapy was also targeted more frequently. These data confirm a reduction in the time to optimal antibiotic therapy, as previously described [9, 30–32]. Despite being a cornerstone of stewardship programs, considered safe and recommended by guidelines [14, 24], de-escalation can be as low as 16% in critically ill patients [32]. In our cohort, spectrum reduction was performed in most cases before definitive culture results and antibiogram availability, within the time window proposed by recommendations [26]. Early de-escalation was performed to a lesser extent in *E. faecium*, polymicrobial, or nosocomial BSI and in patients with inappropriate empiric therapy. Similarly, non-targeted therapy after MALDI-TOF occurred mainly in patients in shock or with BSI with non-faecalis enterococci, polymicrobial, later episodes or from gastro-intestinal source. The presence of multidrug-resistant or non-fermenting Gram-negative bacteria, negative cultures, narrow-spectrum antibiotic therapy, or clinical worsening are some other reasons previously reported not to de-escalate [24].

Consequences of de-escalation have shown conflicting results in literature [33, 34]. We observed a reduction in the LOS in patients where de-escalation took place after correct identification and in all patients whose antibiotic therapy was targeted after MALDI-TOF. A non-significant reduction in short and long-term mortality was observed in both scenarios, but it was not relevant in multivariate analysis.

Regarding MALDI-TOF performance, an identification at the genus level was possible in 87% and at the species level in 73%, overall. Particularly in monomicrobial BSI, *Enterococcus* was identified in 93% of isolates, in 87% and 73% at the genus and species level, respectively. Irrespective of polymicrobial BSI, identification rate of non-faecalis non-faecium enterococci was low, suggesting lower reliability for these strains. Overall, accuracy was slightly lower than in previous enterococcal series [22] and comparable to the global rate of identification of MALDI-TOF, which varies between 76 and 99%, regardless the approach to microbe identification [28]. However, most misidentifications and enterococci with a score < 1.7 were subsequently identified from colonies recovered from subcultures, which likely took place earlier than identification by traditional methods. Noteworthy, during the study period, the protein extraction was done directly on the MALDI-TOF microplate, which likely explains the need of a second round of MALDI-TOF identification with protein extraction done in tubes, with centrifugation steps. Consequently, we have now moved to a systematic protein extraction in tubes, which provides better scores, despite being slightly longer (30 min). Our results must be interpreted considering data collection started in 2010. MALDI-TOF accuracy has progressively evolved since its implementation, which is supported by the better performance we have observed during the most recent time period of the study. This improvement is due to (1) update and enrichment of libraries, (2) dedicated libraries for identification from BC (Bruker Daltonics, Bremen, Germany) allowing an identification with a score ≥ 1.8 instead of ≥ 2, (3) improvement of enrichment and purification methods/kits from positive BC and (4) laboratory automation [35, 36]. 

Thirty-day mortality in E-BSI was high (16%), but slightly lower than previously reported [5–10]. Risk factors independently associated with death were ICU admission, immunosuppression, cirrhosis, endocarditis, catheter-related, gastrointestinal source, primary and polymicrobial BSI. The higher LOS [5] and short and long-term mortality rates [5, 10, 20, 37, 38] observed in *E. faecium* compared to *E. faecalis* have already been described, but *E. faecium* was not associated with mortality in multivariate analysis. This suggests that it is likely not an issue of higher virulence, but possibly host-related confounders (higher prevalence of nosocomial cases and later acquisition, gastrointestinal source, solid malignancy and immunosuppression – last both not significant), and a higher proportion of inappropriate empiric treatment. Inadequacy was more frequent in *E. faecium* BSI due to a rarer empiric use of vancomycin in our setting of very low prevalence of methicillin-resistant *Staphylococcus aureus*. Poor long-term mortality observed was consistent with a recent large series of BSI [39] and, particularly, E-BSI (5-year survival of 24%) [40]. As expected, comorbidities were the most relevant contributors in multivariate analysis.

Patients with polymicrobial BSI had higher mortality, but the literature is inconsistent [41–43]. The prevalence of 39% was similar to previous series (21–44%) [6, 9, 10, 37, 40], mostly due to Gram-negative bacteria. It is known that *Enterococcus* is often initially not directly identified by MALDI-TOF because of its lower growth rate, which explains its lower reliability. Therefore, concomitant Gram stain microscopy is critical [32]. 

The high proportion of nosocomial infections with a late median day for BSI was expected, particularly with *E. faecium* [3, 5, 8, 10, 37] due to its intrinsic resistance, previous antibiotic exposure with a change in intestinal flora, accumulation of complications during hospitalization, and colonization through contact with surfaces.

Regarding patients characteristics, our cohort was similar to previous ones, with a majority of male patients who were old, frail, and polymorbid, particularly with solid malignancy [5, 6, 8, 37]. As a low-virulence bacteria, septic shock occurred in a minority of patients [8, 9]. Gastrointestinal and urinary tract sources are typically the most common, particularly for *E. faecium* and *E. faecalis*, respectively [5, 8–10, 37]. Primary BSI completed the top three of the most common sources [5, 8–10, 37].

Our study has limitations. Even if population characteristics were similar to previous series, and the proportion between enterococci strains was similar in excluded patients, a selection bias may be present due to the analysis exclusively of episodes where ID consultancy took place. On the one hand, ID consultation on E-BSI management improves outcomes [8, 38]. On the other hand, mortality may be underestimated due to exclusion of palliative patients or episodes occurred in the ICU or ID ward where it was not requested.

Data that may interfere with prognosis were not available: duration and definitive antibiotic therapy, source control, clinical or microbiological cure, precise time to effective therapy, antifungals, other risk factors like devices or prior antibiotics, prognostic scores, or ID propositions not applied by clinicians in charge.

Regarding polymicrobial BSI with identification of a second strain of enterococci, we decided to consider the first one identified for analysis. Still, it was a rare finding (6% of all BSI).

We decided to adapt a ranking of antibiotics previously proposed [25], considering the spectrum as the main driver (Tables S1 and S2). There is an inconsistency between ranks used to guide de-escalation in previous studies, highlighting the difficulty of ranking antibiotic spectra, particularly using different classes of antibiotics [24]. Ideally, each classification system should be adapted to its setting, considering also the risk of bacterial selection and its impact on the population [24].

## Conclusion

E-BSI, particularly caused by *E. faecium*, are associated with frequent inappropriate antibiotic therapy and poor short- and long-term outcomes. MALDI-TOF is a reliable tool known for shortening the time to bacterial identification. This taxonomic information is associated with a typical antibiotic susceptibility pattern that helps predicting the appropriate antibiotic treatment. Thus, we observed a significant clinical impact of MALDI-TOF identification on spectrum correction and de-escalation. Earlier appropriate antibiotic therapy improves the LOS and survival, and earlier de-escalation might reduce the risk of antimicrobial resistance.

## Electronic supplementary material

Below is the link to the electronic supplementary material.

Supplementary Material 1

Supplementary Material 2

Supplementary Material 3

Supplementary Material 4

Supplementary Material 5

## Data Availability

No datasets were generated or analysed during the current study.

## Abbreviations

BC: Blood cultures
BSI: Bloodstream infections
E-BSI: *Enterococcus* spp. bloodstream infection
ID: Infectious disease
LOS: Length of stay
MALDI-TOF: Matrix-assisted laser desorption/ionization time-of-flight mass spectrometry
