# Supplementary material for: Impact of rapid identification by MALDI-TOF MS from positive blood cultures in Enterococcus spp. bloodstream infections
Source: Eur J Clin Microbiol Infect Dis. 2025 Mar 8;44(5):1185–96. doi: 10.1007/s10096-025-05084-x (PMC12062115; doi:10.1007/s10096-025-05084-x)
Supplement: Supplementary file 1 — Supplementary Material 1 [file 10096_2025_5084_MOESM1_ESM.docx]

| ***Enterococcus faecalis / Enterococcus faecium* amoxicillin-sensitive/ *Enterococcus* *casseliflavus* amoxicillin-sensitive, vancomycin-resistant / *Enterococcus* *dispar* amoxicillin-sensitive, vancomycin-resistant** | | | | | | | | | | | |
| --- | --- | --- | --- | --- | --- | --- | --- | --- | --- | --- | --- |
| **Inappropriate antibiotic therapy** | | | | | | **Appropriate antibiotic therapy** | | | | | |
| **Spectrum broadening** | | **De-escalation** | | **No change** | | **Spectrum broadening** | | **De-escalation** | | **No change** | |
| **Before MALDI-TOF** | **After MALDI-TOF** | **Before MALDI-TOF** | **After MALDI-TOF** | **Before MALDI-TOF** | **After MALDI-TOF** | **Before MALDI-TOF** | **After MALDI-TOF** | **Before MALDI-TOF** | **After MALDI-TOF** | **Before MALDI-TOF** | **After MALDI-TOF** |
| 0 | amoxicillin | ceftriaxone | amoxicillin | ceftriaxone | ceftriaxone | penicillin | amoxicillin | vancomycin | amoxicillin | vancomycin + (rifampicin) | vancomycin |
| ceftriaxone | pip.-tazobactam | ceftriaxone | amoxicillin- clavulanate |  |  | amoxicillin- clavulanate | amoxicillin + ceftriaxone | amoxicillin- clavulanate | penicillin | amoxicillin- clavulanate | amoxicillin- clavulanate |
| ceftriaxone | ceftriaxone + amoxicillin | ceftriaxone | amoxicillin + vancomycin |  |  | amoxicillin- clavulanate | amoxicillin- clavulanate + ceftriaxone | amoxicillin- clavulanate | amoxicillin | pip.-tazobactam | pip.-tazobactam |
| ceftriaxone | ceftriaxone + vancomycin | ceftriaxone clarithromycin | amoxicillin + (gentamicin) |  |  | amoxicillin- clavulanate | amoxicillin + ciprofloxacin | amoxicillin- clavulanate | amoxicillin + (gentamicin) | pip.-tazobactam | pip.-tazobactam + (gentamicin) |
| cefepime | pip.-tazobactam | ceftriaxone + metronidazol | amoxicillin + (gentamicin) |  |  | amoxicillin- clavulanate | pip.-tazobactam | ceftriaxone + vancomycin | amoxicillin- clavulanate | pip.-tazobactam | pip.-tazobactam + (tobramycin) |
| ertapenem | ertapenem + vancomycin | ceftriaxone + metronidazol | amoxicillin- clavulanate |  |  | pip.-tazobactam | imipenem + (gentamicin) | pip.-tazobactam | amoxicillin | imipenem | imipenem |
|  |  | cefepime | vancomycin |  |  | pip.-tazobactam | pip.-tazobactam + vancomycin + (gentamicin) | pip.-tazobactam | amoxicillin + (gentamicin) |  |  |
|  |  | ciprofloxacin | amoxicillin |  |  | pip.-tazobactam | pip.-tazobactam + clindamycin | pip.-tazobactam | amoxicillin + ceftriaxone |  |  |
|  |  | ertapenem + vancomycin | amoxicillin |  |  | imipenem | imipenem + vancomycin | pip.-tazobactam | amoxicillin- clavulanate |  |  |
|  |  |  |  |  |  |  |  | pip.-tazobactam | amoxicillin- clavulanate + (gentamicin) |  |  |
|  |  |  |  |  |  |  |  | pip.-tazobactam | amoxicillin- clavulanate + vancomycin |  |  |
|  |  |  |  |  |  |  |  | pip.-tazobactam | vancomycin |  |  |
|  |  |  |  |  |  |  |  | ceftolozane-tazobactam + vancomycin + amikacin | ceftolozane-tazobactam + vancomycin |  |  |
|  |  |  |  |  |  |  |  | imipenem | pip.-tazobactam |  |  |
|  |  |  |  |  |  |  |  | meropenem + vancomycin + amikacin | meropenem + vancomycin |  |  |

**Table S1** – Classification of antibiotic therapy decisions according to spectra on *Enterococcus faecalis;* or *Enterococcus faecium* amoxicillin-sensitive, vancomycin-sensitive; or *Enterococcus* *casseliflavus* amoxicillin-sensitive, vancomycin-resistant; or *Enterococcus* *dispar* amoxicillin-sensitive, vancomycin-resistant bloodstream infections. All the decisions analysed during data collection and respective classifications are presented in this table.

Abbreviations: MALDI-TOF, Matrix-assisted laser desorption/ionization time-of-flight mass spectrometry; pip.-tazobactam, piperacillin-tazobactam.
