# Supplementary material for: Impact of rapid identification by MALDI-TOF MS from positive blood cultures in Enterococcus spp. bloodstream infections
Source: Eur J Clin Microbiol Infect Dis. 2025 Mar 8;44(5):1185–96. doi: 10.1007/s10096-025-05084-x (PMC12062115; doi:10.1007/s10096-025-05084-x)
Supplement: Supplementary file 2 — Supplementary Material 2 [file 10096_2025_5084_MOESM2_ESM.docx]

| ***Enterococcus faecium* amoxicillin-resistant, vancomycin-sensitive** | | | | | | | | | | | |
| --- | --- | --- | --- | --- | --- | --- | --- | --- | --- | --- | --- |
| **Inappropriate antibiotic therapy** | | | | | | **Appropriate antibiotic therapy** | | | | | |
| **Spectrum broadening** | | **De-escalation** | | **No change** | | **Spectrum broadening** | | **De-escalation** | | **No change** | |
| **Before MALDI-TOF** | **After MALDI-TOF** | **Before MALDI-TOF** | **After MALDI-TOF** | **Before MALDI-TOF** | **After MALDI-TOF** | **Before MALDI-TOF** | **After MALDI-TOF** | **Before MALDI-TOF** | **After MALDI-TOF** | **Before MALDI-TOF** | **After MALDI-TOF** |
| 0 | vancomycin | ceftriaxone | vancomycin + (gentamicin) | ceftriaxone | ceftriaxone | vancomycin | pip.-tazobactam + vancomycin | pip.-tazobactam + vancomycin | vancomycin | vancomycin + (rifampicin) | vancomycin |
| cefazoline | cefazoline + daptomycin | ceftriaxone + clarithromycin | vancomycin + (gentamicin) |  |  | pip.-tazobactam + vancomycin | pip.-tazobactam + daptomycin | ciprofloxacine+ clindamycine + vancomycin | ciprofloxacine+ vancomycin | vancomycin + (gentamicin + rifampicin) | vancomycin + (gentamicin + rifampicin) |
| ceftriaxone | ceftriaxone + vancomycin | ceftriaxone | vancomycin +  amoxicillin |  |  |  |  | ceftolozane-tazobactam + vancomycin + amikacin | ceftolozane-tazobactam + vancomycin | imipenem + vancomycin | imipenem + vancomycin |
| ceftriaxone + metronidazole | ceftriaxone + metronidazole + vancomycin | cefepime | vancomycin + (gentamicin) |  |  |  |  | meropenem + vancomycin | vancomycin |  |  |
| cefepime | cefepime + vancomycin | pip.-tazobactam | vancomycin |  |  |  |  | meropenem + vancomycin + amikacin | meropenem + vancomycin |  |  |
| cefepime | pip.-tazobactam + vancomycin | pip.-tazobactam | ceftriaxone + vancomycin |  |  |  |  |  |  |  |  |
| ciprofloxacin + metronidazole | ceftriaxone + metronidazole + vancomycin | pip.-tazobactam | amoxicillin-clavulanate + vancomycin |  |  |  |  |  |  |  |  |
| pip.-tazobactam | pip.-tazobactam + vancomycin | pip.-tazobactam | amoxicillin + daptomycin |  |  |  |  |  |  |  |  |
| pip.-tazobactam | ertapenem + vancomycin | meropenem | pip.-tazobactam + vancomycin |  |  |  |  |  |  |  |  |
| ertapenem | ertapenem + vancomycin |  |  |  |  |  |  |  |  |  |  |
| meropenem | meropenem + vancomycin |  |  |  |  |  |  |  |  |  |  |

**Table S2** – Classification of antibiotic therapy decisions according to spectra on *Enterococcus faecium* amoxicillin-resistant, vancomycin-sensitive bloodstream infections. All the decisions analysed during data collection and respective classifications are presented in this table).

Abbreviations: MALDI-TOF, Matrix-assisted laser desorption/ionization time-of-flight mass spectrometry; pip.-tazobactam, piperacillin-tazobactam.
