# Supplementary material for: Impact of rapid identification by MALDI-TOF MS from positive blood cultures in Enterococcus spp. bloodstream infections
Source: Eur J Clin Microbiol Infect Dis. 2025 Mar 8;44(5):1185–96. doi: 10.1007/s10096-025-05084-x (PMC12062115; doi:10.1007/s10096-025-05084-x)
Supplement: Supplementary file 3 — Supplementary Material 3 [file 10096_2025_5084_MOESM3_ESM.docx]

| ***Non-faecalis non-faecium enterococci*** | **N = 15 (%)** |
| --- | --- |
| *E. avium* | 6 (40.0) |
| *E. casseliflavus* | 3 (20.0) |
| *E. durans* | 2 (13.3) |
| *E. hirae* | 2 (13.3) |
| *E. thailandicus* | 1 (6.7) |
| *E. dispar* | 1 (6.7) |

**Table S3 –** Prevalence of non-faecalis non-faecium enterococci isolates.
