# Supplementary material for: Impact of rapid identification by MALDI-TOF MS from positive blood cultures in Enterococcus spp. bloodstream infections
Source: Eur J Clin Microbiol Infect Dis. 2025 Mar 8;44(5):1185–96. doi: 10.1007/s10096-025-05084-x (PMC12062115; doi:10.1007/s10096-025-05084-x)
Supplement: Supplementary file 4 — Supplementary Material 4 [file 10096_2025_5084_MOESM4_ESM.docx]

| **MT *Enterococcus* spp.**  **identification score ≥ 2** | **n=177** | | **De-escalation**  **n (%)**  **n=62 (35.0)** | ***p*-value** | **Targeted antibiotic therapy**  **n (%)**  **n=59 (33.3)** | ***p*-value** |
| --- | --- | --- | --- | --- | --- | --- |
| **Immunosuppression** | No | 117 | 42 (35.9) | 0.863 | 41 (35.0) | 0.613 |
|  | Yes | 60 | 20 (32.3) |  | 18 (30.0) |  |
| **Septic shock** | No | 157 | 55 (35.0) | 1.000 | 58 (36.9) | **0.004** |
|  | Yes | 20 | 7 (35.0) |  | 1 (5.0) |  |
| **Intensive Care Unit admission** | No | 144 | 50 (34.7) | 1.000 | 53 (36.8) | 0.065 |
|  | Yes | 33 | 12 (36.4) |  | 6 (18.2) |  |
| **Clinical source of BSI** |  | |  | |  | |
| **Gastrointestinal tract** | No | 110 | 47 (42.7) | **0.010** | 51 (46.4) | **<0.001** |
|  | Yes | 67 | 15 (22.4) |  | 8 (11.9) |  |
| **Urinary tract** | No | 149 | 47 (31.5) | **0.043** | 45 (30.2) | 0.069 |
|  | Yes | 28 | 15 (53.6) |  | 14 (50.0) |  |
| **Catheter-related** | No | 154 | 55 (35.7) | 0.794 | 49 (31.8) | 0.385 |
|  | Yes | 23 | 7 (30.4) |  | 10 (43.5) |  |
| **Other source*** | No | 158 | 57 (36.1) | 0.457 | 53 (33.5) | 1.000 |
|  | Yes | 19 | 5 (26.3) |  | 6 (31.6) |  |
| **Endocarditis** | No | 165 | 55 (33.3) | 0.115 | 50 (30.3) | **0.004** |
|  | Yes | 12 | 7 (58.3) |  | 9 (75.0) |  |
| **Unknown (primary)** | No | 149 | 49 (32.9) | 0.245 | 47 (31.5) | 0.344 |
|  | Yes | 28 | 13 (46.4) |  | 12 (42.9) |  |
| **Setting of BSI acquisition** |  | |  | |  | |
| **Community-acquired BSI** | No | 156 | 53 (34.0) | 0.577 | 49 (31.4) | 0.218 |
|  | Yes | 21 | 9 (42.9) |  | 10 (47.6) |  |
| **Nosocomial BSI** | No | 58 | 28 (48.3) | **0.016** | 23 (39.7) | 0.282 |
|  | Yes | 119 | 34 (28.6) |  | 36 (30.3) |  |
| **Healthcare-associated BSI** | No | 140 | 43 (30.7) | **0.032** | 46 (32.9) | 0.948 |
|  | Yes | 37 | 19 (51.4) |  | 13 (35.1) |  |

**Table S4** – Prevalence of de-escalation and targeted antibiotic therapy after MALDI-TOF according to clinical characteristics of population.

Abbreviations: BSI, bloodstream infection; MALDI-TOF, Matrix-assisted laser desorption/ionization time-of-flight mass spectrometry;

*“Other source” includes surgical site, soft tissue, osteoarticular or other endovascular sources.
