# Supplementary material for: Impact of rapid identification by MALDI-TOF MS from positive blood cultures in Enterococcus spp. bloodstream infections
Source: Eur J Clin Microbiol Infect Dis. 2025 Mar 8;44(5):1185–96. doi: 10.1007/s10096-025-05084-x (PMC12062115; doi:10.1007/s10096-025-05084-x)
Supplement: Supplementary file 5 — Supplementary Material 5 [file 10096_2025_5084_MOESM5_ESM.docx]

| **Characteristics of patients** |  | **N=267** | **30-day mortality**  **n (%)**  **43 (16.1)** | ***p*-value** | **1-year mortality**  **n (%)**  **116 (43.4)** | ***p*-value** |
| --- | --- | --- | --- | --- | --- | --- |
| **Comorbidities** |  |  |  |  |  |  |
| **Cardiomyopathy** |  | 85 | 10 (11.8) |  | 31 (36.5) |  |
| **Chronic pulmonary disease** |  | 41 | 8 (19.5) |  | 19 (46.3) |  |
| **Cirrhosis** |  | 24 | 7 (29.2) |  | 16 (66.7) |  |
| **Chronic kidney disease (stage III-V)*** |  | 48 | 6 (12.8) |  | 27 (57.4) |  |
| **Dialysis** |  | 15 | 2 (13.3) |  | 8 (53.3) |  |
| **Diabetes** |  | 60 | 10 (16.7) |  | 31 (51.7) |  |
| **Immunosuppression** | No | 188 | 26 (13.8) | 0.168 | 67 (35.6) | **<0.001** |
|  | Yes | 79 | 17 (21.5) |  | 49 (62.0) |  |
| **Transplantation** |  | 21 | 5 (23.8) |  | 14 (66.7) |  |
| **Haematological malignancy** |  | 28 | 8 (28.6) |  | 20 (71.4) |  |
| **Solid malignancy**** |  | 108 | 18 (16.7) |  | 59 (54.6) |  |
| **Gastrointestinal tract** |  | 64 | 14 (21.9) |  | 37 (57.8) |  |
| **Urinary tract** |  | 24 | 1 ( 4.2) |  | 9 (37.5) |  |
| **Other** |  | 30 | 5 (16.7) |  | 19 (63.3) |  |
| **Septic shock** | No | 234 | 32 (13.7) | **0.009** | 97 (41.5) | 0.118 |
|  | Yes | 33 | 11 (33.3) |  | 19 (57.6) |  |
| **ICU admission** | No | 224 | 28 (12.5) | **0.001** | 94 (42.0) | 0.344 |
|  | Yes | 43 | 15 (34.9) |  | 22 (51.2) |  |
| **Clinical source of BSI** |  |  |  |  |  |  |
| **Gastrointestinal tract** |  | 103 | 21 (20.4) |  | 54 (52.4) |  |
| **Urinary tract** |  | 40 | 2 (5.0) |  | 17 (42.5) |  |
| **Catheter-related** |  | 34 | 7 (20.6) |  | 13 (38.2) |  |
| **Other***** |  | 29 | 2 (6.9) |  | 9 (31.0) |  |
| **Endocarditis** |  | 18 | 3 (16.7) |  | 6 (33.3) |  |
| **Unknown (primary)** |  | 43 | 8 (18.6) |  | 17 (39.5) |  |
| **Setting of BSI acquisition** |  |  |  |  |  |  |
| **Community-acquired BSI** |  | 36 | 3 (8.3) | **0.045** | 10 (27.8) | 0.124 |
| **Nosocomial BSI** |  | 180 | 36 (20.0) |  | 83 (46.1) |  |
| **Healthcare-associated BSI** |  | 51 | 4 (7.8) |  | 23 (45.1) |  |
| **Polymicrobial BSI** | No | 163 | 19 (11.7) | **0.021** | 71 (43.6) | 1.000 |
|  | Yes | 104 | 24 (23.1) |  | 45 (43.3) |  |
| **Non-faecalis non-faecium BSI** |  | 15 | 3 (20) |  | 6 (40) |  |

**Table S5 –** Thirty-day and 1-year mortality according to clinical characteristics of population.

Abbreviations: BSI, bloodstream infection; ICU, Intensive Care Unit.

*Chronic kidney injury according to KDIGO classification;

**Cured or active malignancy were considered;

***“Other source” includes surgical site, soft tissue, osteoarticular or other endovascular sources.
